# Treatment of an Extensive Maxillary Cyst Using Nasal Airway and Balloon Catheter Devices

**DOI:** 10.1155/2014/216828

**Published:** 2014-04-06

**Authors:** Atsushi Kasamatsu, Chonji Fukumoto, Morihiro Higo, Yosuke Endo-Sakamoto, Katsunori Ogawara, Masashi Shiiba, Hideki Tanzawa, Katsuhiro Uzawa

**Affiliations:** ^1^Department of Dentistry and Oral-Maxillofacial Surgery, Chiba University Hospital, 1-8-1 Inohana, Chuo-ku, Chiba 260-8670, Japan; ^2^Department of Clinical Molecular Biology, Graduate School of Medicine, Chiba University, 1-8-1 Inohana, Chuo-ku, Chiba 260-8670, Japan; ^3^Department of Clinical Oncology, Graduate School of Medicine, Chiba University, 1-8-1 Inohana, Chuo-ku, Chiba 260-8670, Japan

## Abstract

*Introduction*. Large maxillary cysts occasionally expand into the maxilla and erode the maxillary sinus and nasal cavity. The Caldwell-Luc procedure is the recommended treatment for large maxillary sinus cysts. However, it is hard to preserve the nasal space in the case of large maxillary sinus cysts that penetrate into the nasal cavity. *Methods*. A 22-year-old man who had large maxillary sinus cysts was referred to our department for a surgical treatment. After removing the cyst from the maxillary sinus using the Caldwell-Luc procedure, we used nasal airway and balloon catheter devices to preserve the space of the inferior nasal meatus and maxillary sinus. These devices were removed 10 days postoperatively. Insertion and removal of both devices were simple and painless. *Findings*. The nasal airway and balloon catheter devices were useful for performing maxillary sinus surgery to remove large cysts. Our method was satisfactorily safe and was an effective minimally invasive treatment that preserved the space of the inferior nasal meatus and maxillary sinus.

## 1. Introduction

Among large maxillary cysts that expand to the maxillary sinus, dentigerous cysts, and radicular cysts are two main odontogenic cysts [1]. In the oral and maxillofacial regions, cyst enucleation combined with the Caldwell-Luc procedure [2, 3], which has been the mainstay of maxillary sinus surgery over the past century, was adopted for their treatment [4]. In this report, we describe a new method to treat maxillary cysts that have expanded into the maxilla, the maxillary sinus, and the nasal cavity using nasal airway and balloon catheter devices after the Caldwell-Luc procedure.

## 2. Case Presentation

**Figure 1:**
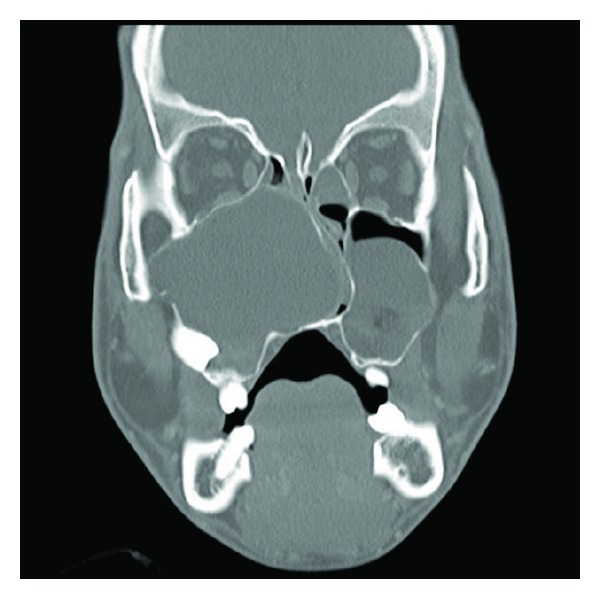
A preoperative coronal computed tomography scan shows a large cystic lesion involving the right maxillary sinus and inferior nasal meatus.

**Figure 2:**
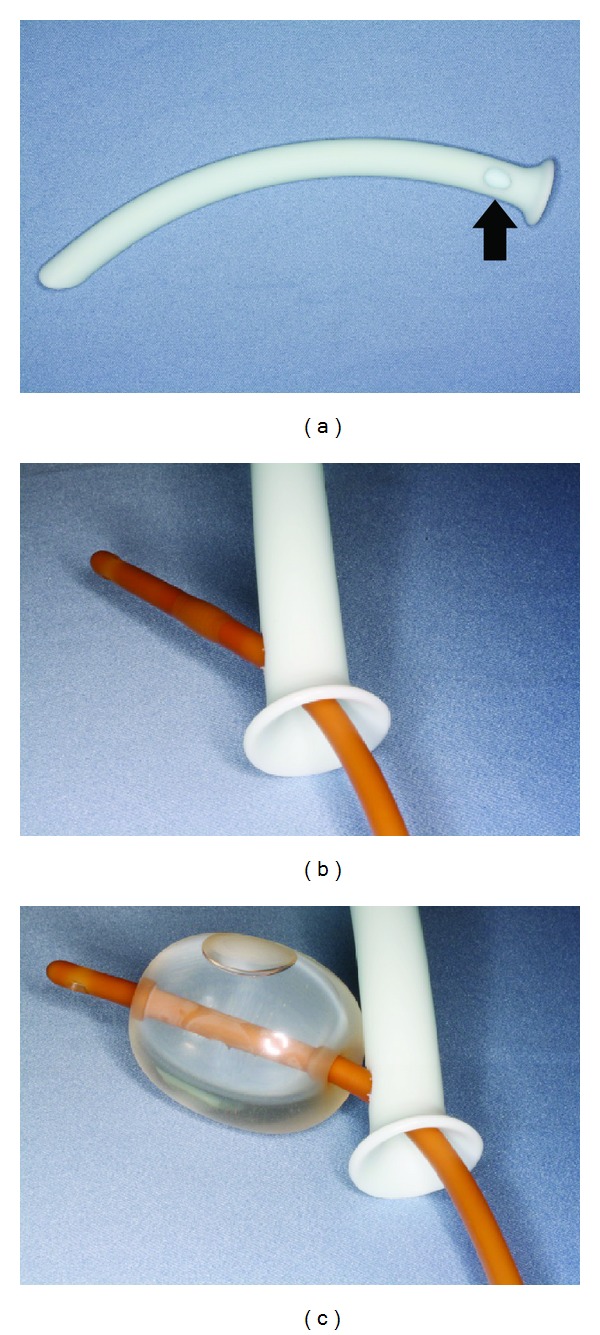
Nasal airway and balloon catheter devices are assembled through the window of the nasal airway (a, b, and c).

**Figure 3:**
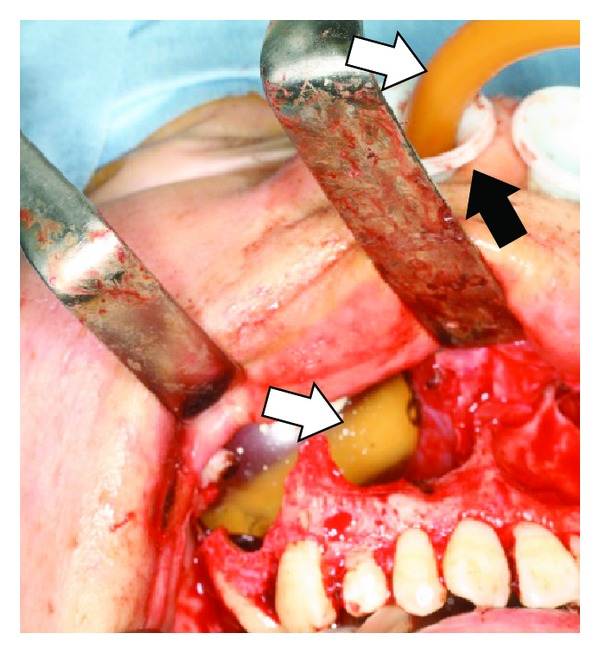
The operative view of the nasal airway (solid arrow) and the balloon catheter (open arrow) devices in the right maxillary sinus.

**Figure 4:**
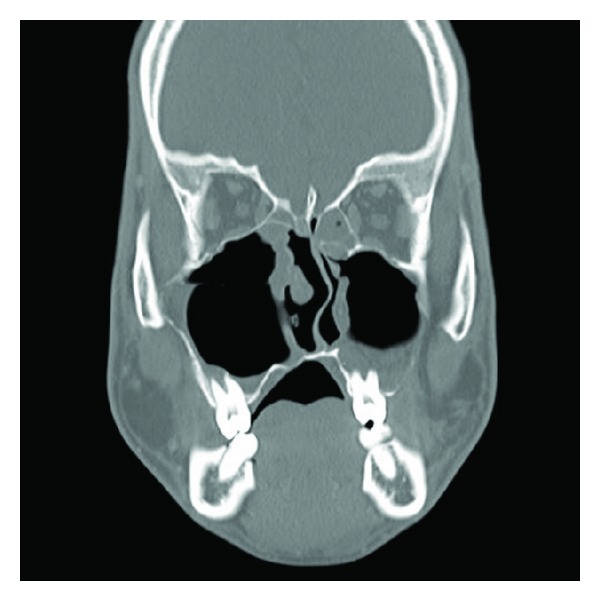
A postoperative coronal computed tomography scan shows that the right maxillary cyst has been removed and obstruction of the inferior nasal meatus has resolved completely.

A 22-year-old man suffering from complete nasal obstruction for the last 5 months was referred to our department. Computed tomography scan showed impacted teeth along with the radiopaque borders in bilateral maxillary sinuses (Figure 1). To remove the large maxillary sinus cysts and preserve the space of the inferior nasal meatus and maxillary sinus, surgical enucleation for the cysts was performed according to the Caldwell-Luc procedure [2–4]. After removing the cysts from the maxillary sinus, we created a nasoantral window in the lateral nasal wall as described previously [2, 3] and a hole at the same position as the nasoantral window in the nasal airway device (Figure 2(a) arrow). The maxillary sinus was packed with the balloon catheter, instead of a gauze tampon, through the lateral hole of the nasal airway (Figures 2(b), 2(c), and 3). These devices were removed 10 days postoperatively. A final diagnosis of dentigerous cyst in the maxillary sinus was made. The postoperative period was unremarkable and the patient is asymptomatic without signs of recurrence 3 years postoperatively (Figure 4).

## 3. Discussion

The balloon catheter approach for maxillary sinus surgery was introduced to reduce mucosal trauma, scarring, and bleeding of the maxillary sinus [5]. Since large maxillary sinus cysts occasionally erode the maxillary medial wall and penetrate into the nasal cavity, the position of the balloon catheter can be unstable in the maxillary sinus. To resolve this problem, we used a nasal airway device combined with a balloon catheter approach after maxillary sinus surgery. The advantages of our combined method are that the nasal airway and balloon catheter devices preserve the space of the inferior nasal meatus and maxillary sinus, both devices are inserted easily, this technique does not add operative time, and both devices can be removed simply and painlessly.

In the current case, no serious adverse events related to the two devices occurred, and no complications have occurred postoperatively (Figure 4). The nasal airway and balloon catheter devices were useful tools to perform maxillary sinus surgery to remove large cysts, especially those that penetrated into the maxillary medial wall. The results indicated that our method is satisfactorily safe and is an effective minimally invasive treatment option for preserving the space of the inferior nasal meatus and maxillary sinus.
